# Single cell analysis reveals intra‐tumour heterogeneity, microenvironment and potential diagnosis markers for clear cell renal cell carcinoma

**DOI:** 10.1002/ctm2.713

**Published:** 2022-05-23

**Authors:** Man Zhang, Wei Zhai, Juju Miao, Xiaomu Cheng, Wenqin Luo, Weichen Song, Jia Wang, Wei‐Qiang Gao

**Affiliations:** ^1^ State Key Laboratory of Oncogenes and Related Genes, Renji‐Med‐X Stem Cell Research Center, Renji Hospital, School of Medicine and School of Biomedical Engineering Shanghai Jiao Tong University, Shanghai, China; ^2^ School of Biomedical Engineering and Med‐X Research Institute Shanghai Jiao Tong University Shanghai China; ^3^ Department of Urology, Renji Hospital, School of Medicine Shanghai Jiao Tong University Shanghai China; ^4^ Shanghai Mental Health Center, School of Medicine Shanghai Jiao Tong University Shanghai China; ^5^ Department of Interventional Radiology Fudan University Shanghai Cancer Center Shanghai China; ^6^ Present address: School of Life Sciences Westlake University Hangzhou China; ^7^ Present address: Department of Colorectal Surgery Fudan University Shanghai Cancer Center Shanghai China

We found specific gene sets and cell clusters that were correlated with the prognosis for clear cell renal cell carcinoma (ccRCC). We also discovered transcription factor regulons and ligand–receptor pairs that might become potential therapeutic targets.

Single‐cell RNA sequencing (scRNA‐seq) has yielded insights into tumour origin or immune composition's effect on clinical outcome in ccRCC,^1^, ^2^ but was limited to characterize potential new targets.

Here, we performed scRNA‐seq on seven patients’ tumours and matched five normal samples (Table S1 and Figure 1A). A total of 37 243 cells were divided into six cell types according to marker genes (Figure 1B). Potential malignant cells were distinguished via copy number variations (CNVs).^3^ Epithelial cells having CNVs were named as 'changed' (Figure 1B). The top five differentially expressed genes (DEGs) were shown (Figure 1E). According to the cell distributions and sample origins (Figure 1C), we found the heterogeneity of tumour epithelial cells was more obvious than other cells. We then used the Harmony algorithm to minimize the batch effect (Figure 1D,F). Myeloid cells appeared more in tumours than in normal samples, indicating their active role in tumourigenesis (Figure 1G). Scoring for genes with different biological functions (Table S2) indicated that hypoxia score was highest in tumour and endothelial cells (Figure S1).

**FIGURE 1:**
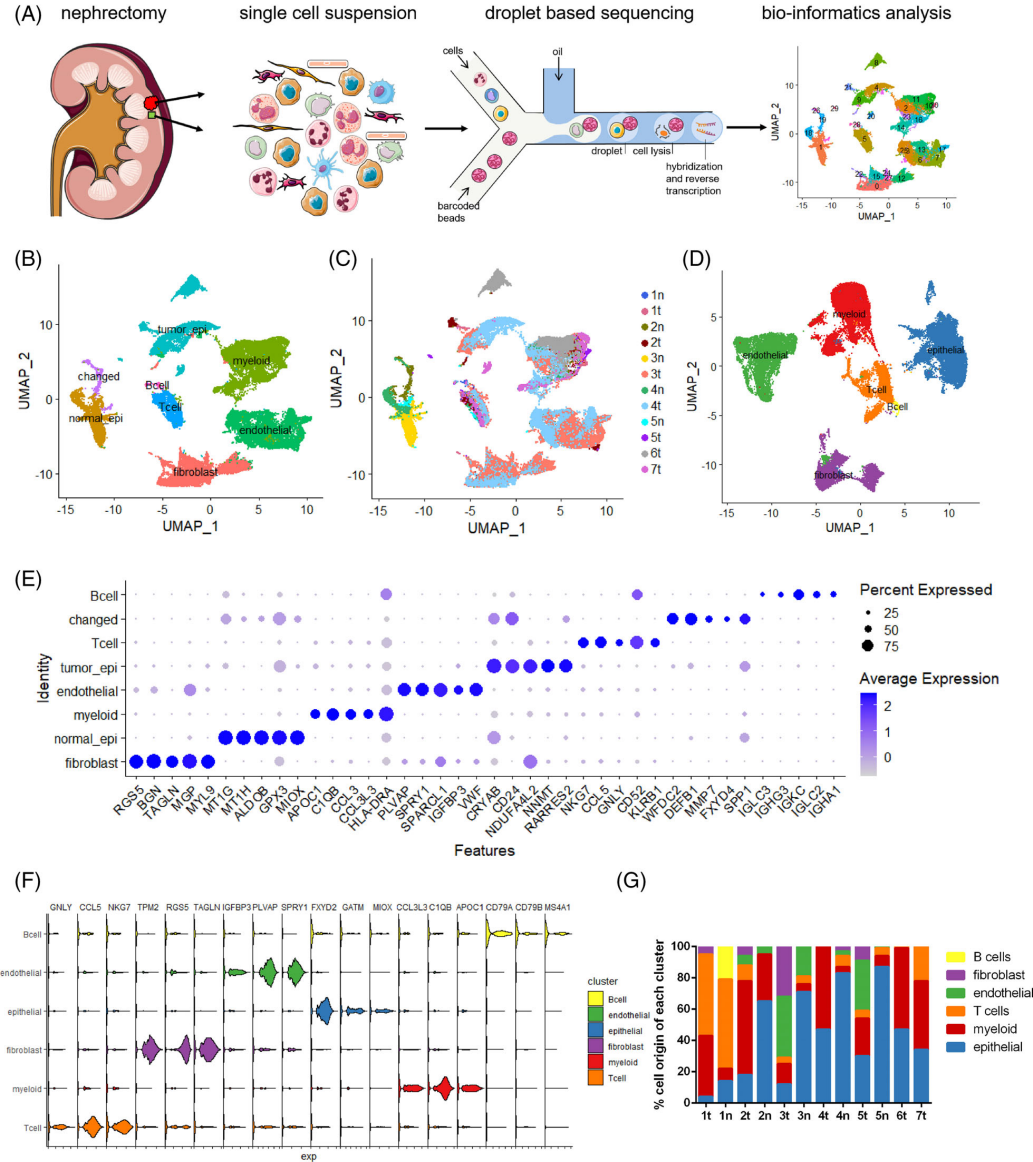
Single cell atlas of ccRCC tumour and normal samples. (A) Workflow for samples. (B) UMAP distribution of major cell types for all the cells that we sequenced. (C) Sample origins. (D) Cell‐type distribution after Harmony treatment. (E) Top five gene markers for each cell type before Harmony treatment. (F) Top three genes markers for each cell type after Harmony treatment. (G) Proportions of cell types in each sample

According to canonical makers (Table S3), epithelial cells were divided into six types (Figure 2A): tumour cell, proximal tubule cell (PT), collecting duct cell (CD), principal cell (PC), podocytes and VCAM1^+^ PT. The ccRCC was thought to originate from VCAM1^+^ PT,^1^ and our data confirmed the existence of this cluster of cells, and these cells were only found in normal samples (Figure S2B). Based on CNVs, epithelial cells were divided into three subpopulations: tumour, normal and 'changed'. Normal cells were mostly PT, while 'changed' cells were comprised CD, PC and VCAM1^+^ PT (Figure 2B,C and Figure S2A).

**FIGURE 2:**
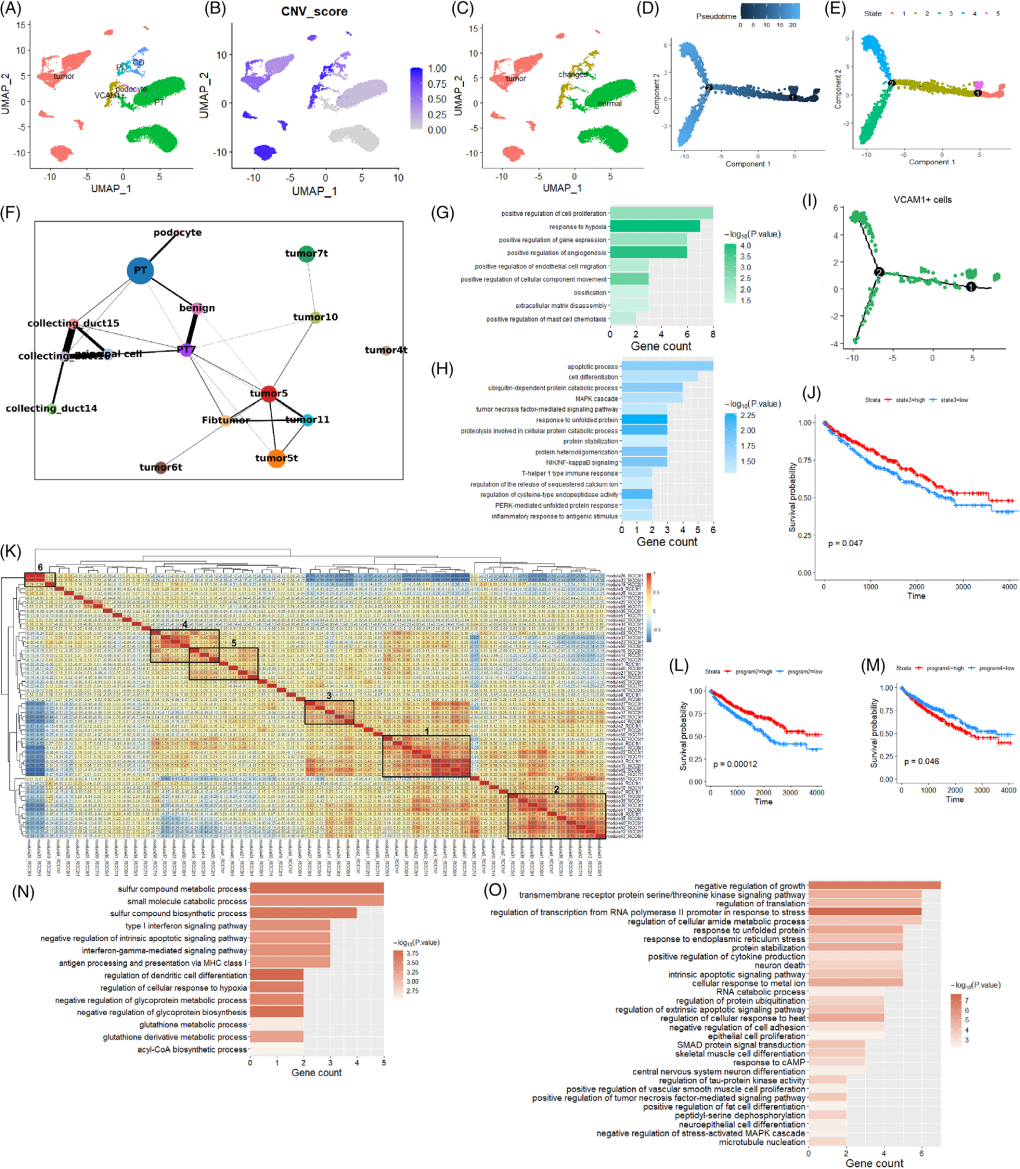
Heterogeneity and dynamics for epithelial cells. (A) Cell types for re‐clustered epithelial cells. CD, collecting duct cell; PC, principal cells; PT, proximal tubule cell; VCAM1^+^, VCAM1 gene positive proximal tubule cell. (B) Inferred gene copy number variation (CNV) score for all epithelial cells. PT cells were used as normal control (grey part at lower right corner). The darker the blue, the greater the variation. (C) According to the CNV score, epithelial cells were divided into three major types: tumour, changed and normal. (D) Pseudotime distribution of all the epithelial cells analysed by Monocle 2. (E) Cell states partitioned by Monocle 2. Different states were distinguished by differentiated expressed gene profiles. (F) PAGA analysis graph for the similarity and transition tendency among different epithelial clusters. PT: normal proximal tubule cells. PT7: VCAM1^+^ proximal tubule cells. Benign: proximal tubule cell cluster whose expression profile was similar to VCAM1^+^ proximal tubule cells but the CNV was normal. Labels containing tumour were different tumour cell clusters. Collecting duct cells had three clusters: 14, 15 and 16. The nearest to collecting duct cells were principal cells. (G, H) GO enrichment analysis for state 3 (green) and state 4 cells (blue). (I) VCAM1^+^ PT cells distribution in pseudotime trajectory. (J) Overall survival results for state 3 genes. (K) Six transcriptional programs were found after NMF analysis. (L, M) Overall survival analysis for program 2 and program 4 gene sets in TCGA cohort. (N, O) GO enrichment analysis for program 2 and program 4 gene sets

To reveal the dynamics of transcriptional profiles of epithelial cells, we applied trajectory analysis (Figure 2D,E). Normal cells are located at the right end of the pseudotime trajectory, while tumour cells are located at the left end, indicating a transition tendency. VCAM1^+^ PT occupied all the branches, suggesting their pluripotency (Figure 2I). PAGA also confirmed VCAM1^+^ PT were most similar to tumour cells (Figure 2F). Monocle2 defined the branches of tumour cells as two 'states' (state 3 and state 4, Figure 2E). State 3 genes functioned in proliferation, hypoxia and angiogenesis, while state 4 genes functioned in stress and immune response (Figure 2G,H and Figure S2C,D and Table S4). High expression of state 3 genes was related to improved overall survival in a TCGA cohort containing 531 patients^4^ (Figure 2J).

NMF^5^ found six tumour epithelial‐specific gene programs (Figure 2K). Highly expressed program 2 was correlated with favourable overall survival, while highly expressed program 4 (Table S5) was correlated with unfavourable survival (Figure 2L,M). Program 2 functioned in interferon signalling and antigen processing (Figure 2N), whereas program 4 functioned in stress response, growth and apoptosis (Figure 2O).

SCENIC^6^ discovered transcription factor regulons differentially expressed in epithelial cells (Figure 3A,B). NR1I3 regulon was normal cell specific, while IRX3 regulon was located mainly in tumour cells (Figure 3C). Co‐expressed regulons clustered together by correlation analysis (Figure S3A). IRX3 level in the tumour was apparently higher than normal samples (Figure 3D). IRX3 was correlated to ccRCC marker CA9 and EMT gene VIM (Figure 3E and Figure S3B). High expression of IRX3 was correlated to unfavourable survival in the TCGA cohort (Figure S3C). We also performed immunofluorescence staining for the seven patients and tissue microarrays containing 340 ccRCC samples. We found IRX3 staining in patient 1t (Figure 3F) and tissue microarrays (Figure 3G), and the positive proportions increased with tumour grade progression (I: 13%, II: 9%, III: 53%). IRX3 had two locations: cytoplasm and nuclei (Figure 3F,G). The patient 1t was diagnosed with lung metastasis after nephrectomy. The metastatic sites had nuclei‐localized IRX3, whereas the primary sites had cytoplasm‐localized IRX3 (Figure 3F). ccRCC cell lines A498 and 769‐P had cytoplasm localization, whereas OSRC‐2, 786‐O and Caki‐1 had nuclei localization (Figure S3D). Location change suggested function change, which needs more investigations. Knockdown of IRX3 significantly decreased the viability, wound healing, migration and invasion ability of OSRC‐2 cells (Figure 3H,I and Figure S3E). IRX3 played a role in ccRCC progression.

**FIGURE 3:**
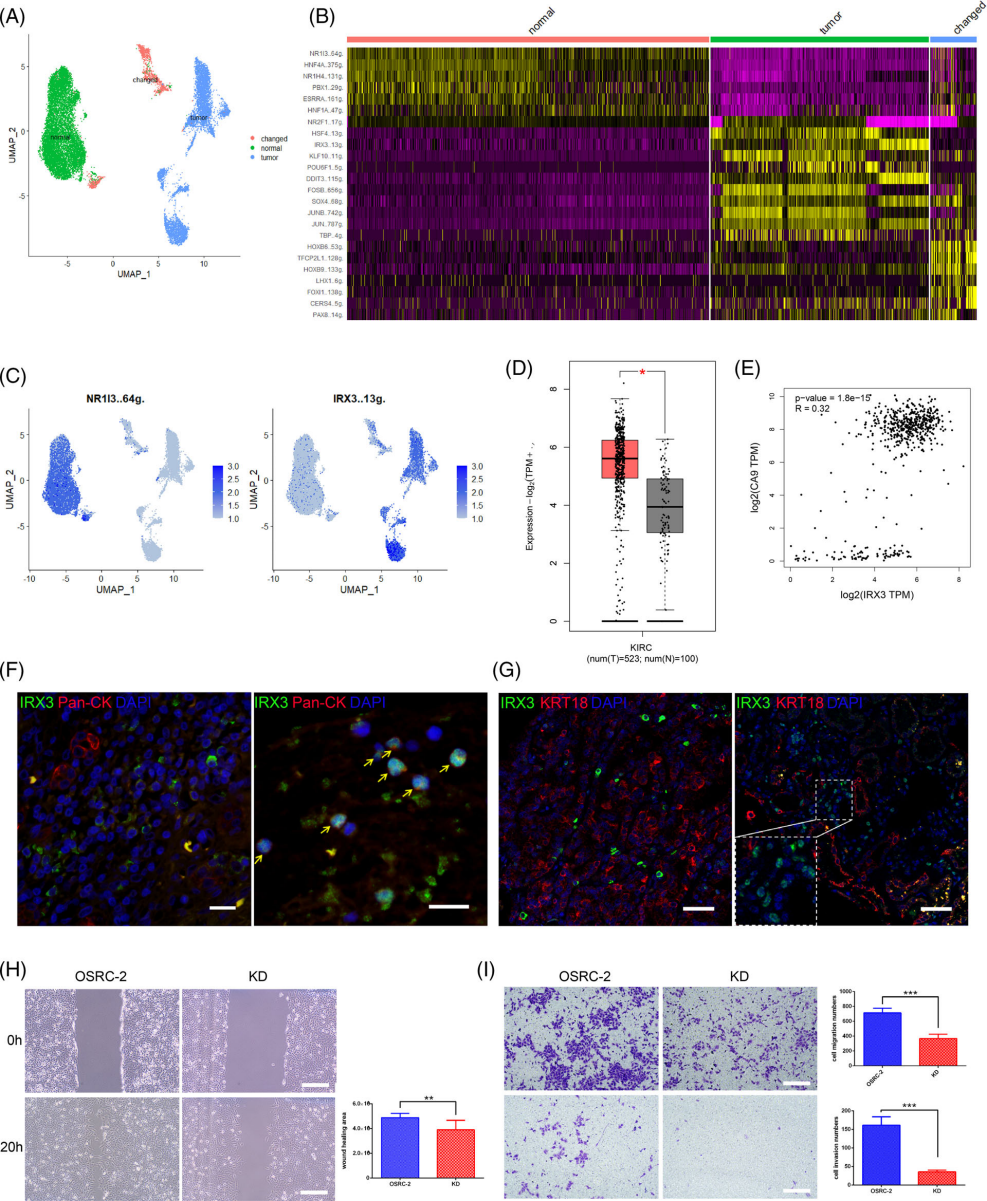
Transcription factor regulons in tumour epithelial cells. (A) UMAP distribution of epithelial cell types: tumour, normal and changed. (B) Top 10 cell‐type‐specific transcriptional factor regulons. (C) Expression levels of normal specific regulon NR1I3 and tumour‐specific regulon IRX3 in all epithelial cells. '..64 g' and '..13 g' means the number of transcription factor target genes. (D) For ccRCC, expression of IRX3 in tumour was significantly higher than in normal samples. Boxplot was made from GEPIA2 online tool based on a ccRCC‐TCGA dataset with 523 tumour samples and 100 normal samples in TCGA database (http://gepia2.cancer‐pku.cn/#index). (E) IRX3 and CA9 had weak correlation. Correlation figure was also made from GEPIA2 online tool. (F) IRX3 expression in primary site (left) and metastatic site (right) of patient 1t. Scale bar: 20 μm. (G) Cytoplasm‐localized (left) and nuclei‐localized (right) IRX3 in ccRCC tissue microarrays. Inset indicates the nuclei localization. Scale bar: 50 μm. (H) Compared to wild type, the wound healing ability of IRX3‐knockdown cells decreased apparently. KD: IRX3 knockdown. Scale bar: 100 μm. (I) Compared to wild type, the migration (upper panel) and invasion (lower panel) ability of IRX3‐knockdown cells decreased significantly. Scale bar: 100 μm

Myeloid cells had 10 clusters (Figure 4A and Figure S4A). We generated a gene signature file of the 10 clusters and used CIBERSORTx^7^ to infer cell‐type proportions in the TCGA cohort. Higher infiltration of C4_C3AR1^+^ macrophages in the tumour was associated with worse survival, while higher infiltration of C3_CD163^+^ macrophages or C8_cDC2 was related to improved survival (Figure 4B). C4_C3AR1^+^ cells expressed tissue‐resident marker CD74 and CD81 and scavenger receptor MSR1, which was an anti‐inflammatory M2 marker. M1 and M2 gene signatures (Table S6) scoring found that C1_IL1B^+^ cells had higher M1 score, while C3_CD163^+^, C5_MSR1^+^ cells had higher M2 scores (Figure 4D). RNA velocity analysis^8^ indicated M1 to M2 transition tendency exist (Figure 4C), and M2‐like cells were mainly from tumour samples based on the sample origins (Figure S4B).

**FIGURE 4:**
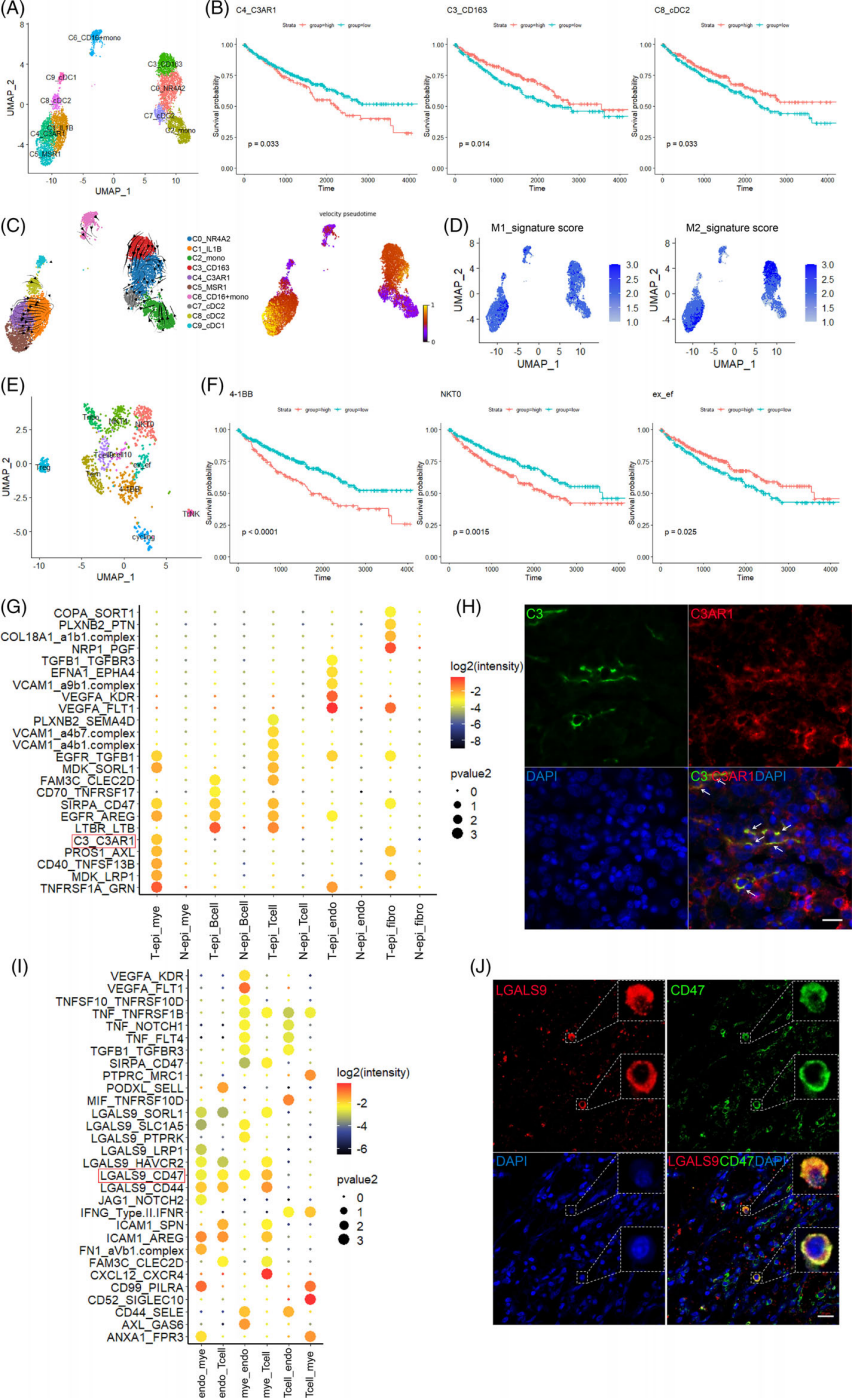
Characteristics of myeloid cells and T cells, cell–cell interactions between different cell types. (A) UMAP distribution of 10 myeloid subclusters. (B) High infiltration of C4_C3AR1 positive cells was related to unfavourable survival in TCGA dataset. High infiltration of C3_CD163 positive cells and C8_cDC2 cluster was associated with favourable overall survival. (C) RNA velocity analysis for myeloid cells. Left: stream plot. Right: pseudotime plot. Both of the plots indicated the transition tendency. (D) M1 and M2 signature scores in myeloid cells. (E) UMAP distribution of T cell subclusters. (F) High infiltration of 4‐1BB positive CD8^+^ T cells and NKT0 cluster was associated with unfavourable overall survival. High infiltration of ex_ef T cells was associated with favourable overall survival. (G) Inferred ligand_receptor interacting pairs between epithelial cells and other cells. endo, endothelial cells; fibro, fibroblasts; mye, myeloid cells and T_epi, tumour epithelial cells. (H) Immunofluorescence staining indicated co‐localization of ligand C3 and receptor C3AR1 in ccRCC samples. Arrows indicates co‐localized cells. Scale bar: 20 μm. (I) ligand_receptor interaction pairs among endothelial cells, myeloid cells and T cells. (J) Immunofluorescence staining indicated co‐localization of ligand LGALS9 and receptor CD47 in ccRCC samples. Scale bar: 20 μm

In subgroups of T cells (Figure 4E), CD8^+^ T cells had three clusters: 4‐1BB+, ex_ef (expressing exhausted makers and effector molecules) and cycling T cells. Higher infiltration of 4‐1BB^+^ T cells or NKT0 cells was related to worse survival, while higher infiltration of ex_ef T cells was related to better survival (Figure 4F). The previous opinion that CD8^+^ T cells predicted bad outcomes^9^ was not accurate, more markers were needed to evaluate the prognosis.

CellPhoneDB inferred interactions between different cells. The strongest interactions appeared between epithelial and myeloid cells (Figure S4C,D and Table S7). Typical crosstalks were listed (Figure 4G,I). Intriguingly, C3_C3AR1 crosstalk happened between tumour epithelial and myeloid cells. The exact roles of complement components C3 and C3AR1 in ccRCC have not been described. C3 and C3AR1 did co‐localize in tumour samples (Figure 4H). LGALS9_CD47 interactions were also obvious (Figure 4I), staining validated this crosstalk (Figure 4J). CD99_PILRA between endothelial and myeloid cells (Figure 4I) suggested a decreasing immune infiltration effect.^10^ LGALS9_HAVCR2/TIM3 also reminded a suppressive impact, suggesting endothelial cells’ immune‐suppression role.

Our studies revealed gene sets and specific macrophage and T cell clusters that can predict prognosis. Potential therapeutic targets such as IRX3 and inhibitory interactions among different cells provided insights for ccRCC therapies.

## CONFLICT OF INTEREST

The authors declare no conflict of interest.

## Supporting information


**Figure S1** The score for all the cell types using gene sets is related to different biological functions. Angiogenesis genes were most abundant in endothelial cells, and then tumour epithelial cells; myeloid cells expressed the strongest fatty acid synthesis gene signatures; glycolysis and oxidative phosphorylation signatures were comparable in tumour epithelial cells, which was beyond our anticipation, as tumour cells had been thought to prefer glycolysis; score of hypoxia genes was highest in tumour epithelial cells, and then endothelial cells.

**Figure S2** (A) infer CNV results for all epithelial clusters with normal proximal tubule cells (NPT) cluster 0 and 6 as normal reference. PC: principal cells, CD: collecting duct cells, Podo: podocytes. inter9 stands for an intermediate cell cluster. (B) Left, Subclustering of all epithelial cells with a higher resolution, which were divided into 25 clusters. Cluster 18 stands for VCAM1^+^ cells. Middle, sample origins for epithelial cells. Right: VCAM1 expression profiles in epithelial cells. (C) Comparison of gene signature scores between state 3 and state 4 cells. ****stands for that *p*‐value of students’ t‐test was less than .0001. (D) Antigen presentation related genes HLA‐DRB5, IL18 and MDK were upregulated in state 4 cells, while angiogenesis related genes VEGFA and PGF, and hypoxia induced gene MT3 were upregulated in state 3 cells.

**Figure S3** (A) Correlation analysis of transcription factor regulons. Co‐upregulated regulons clustered together. (B) IRX3 and EMT gene VIM had correlation in TCGA cohort. (C)Overall survival curve of IRX3 in TCGA cohort containing 528 ccRCC patients. *p*‐score = .048. This figure was obtained from https://www.proteinatlas.org/ENSG00000177508‐IRX3/pathology/renal+cancer/KIRC. (D) Immunofluorescence staining results of 5 ccRCC cell lines. IRX3 located mainly in cytoplasm for A498 and 769‐P, whereas IRX3 mainly located in nuclei of OSRC‐2, 786‐O and Caki‐1. Scale bar: 20μm. (E) Viability validation with CCK8. IRX3 knockdown cell lines were obviously weaker than wildtype.

**Figure S4** (A) Top 5 genes and expression levels in myeloid clusters. (B) Sample origins for all the myeloid cells. (C) Interaction strength between different cells in tumour versus normal samples. T_epi: tumour epithelial cells, N_epi: normal epithelial cells. (D) Circos plot indicates the interaction strengths among tumour epithelial cells versus other cell types, and normal epithelial cells versus other cell types.

Table S1

Table S2

Table S3

Table S4

Table S5

Table S6

Table S7

Supporting information
