# Supplementary figures and images for: Single cell analysis reveals intra‐tumour heterogeneity, microenvironment and potential diagnosis markers for clear cell renal cell carcinoma
Source: Clin Transl Med. 2022 May 23;12(5):e713. doi: 10.1002/ctm2.713 (PMC9126499; doi:10.1002/ctm2.713)

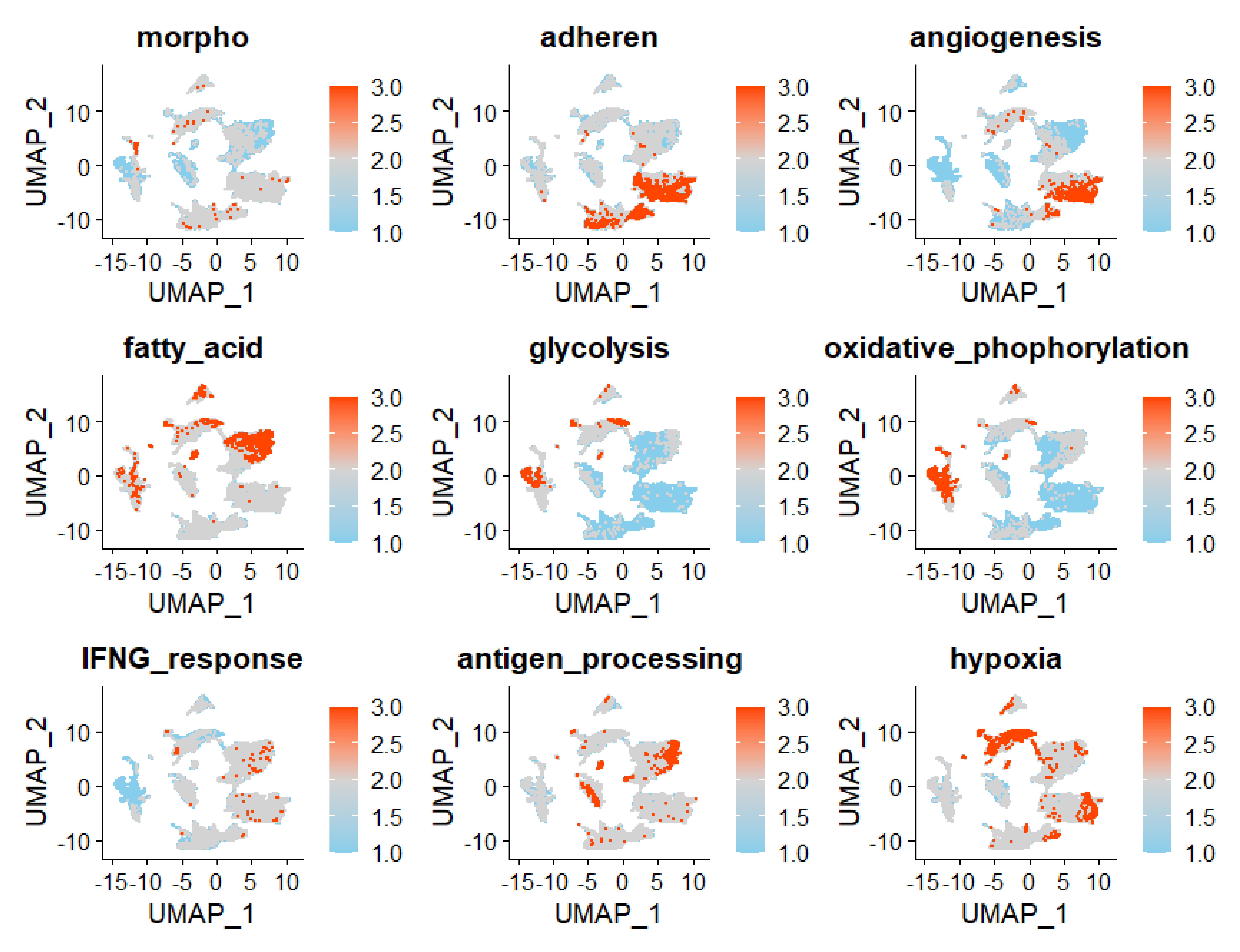

Supplement: Supplementary file 1 — Figure S1 The score for all the cell types using gene sets is related to different biological functions. Angiogenesis genes were most abundant in endothelial cells, and then tumour epithelial cells; myeloid cells expressed the strongest fatty acid synthesis gene signatures; glycolysis and oxidative phosphorylation signatures were comparable in tumour epithelial cells, which was beyond our anticipation, as tumour cells had been thought to prefer glycolysis; score of hypoxia genes was highest in tumour epithelial cells, and then endothelial cells. [file CTM2-12-e713-s011.jpg]

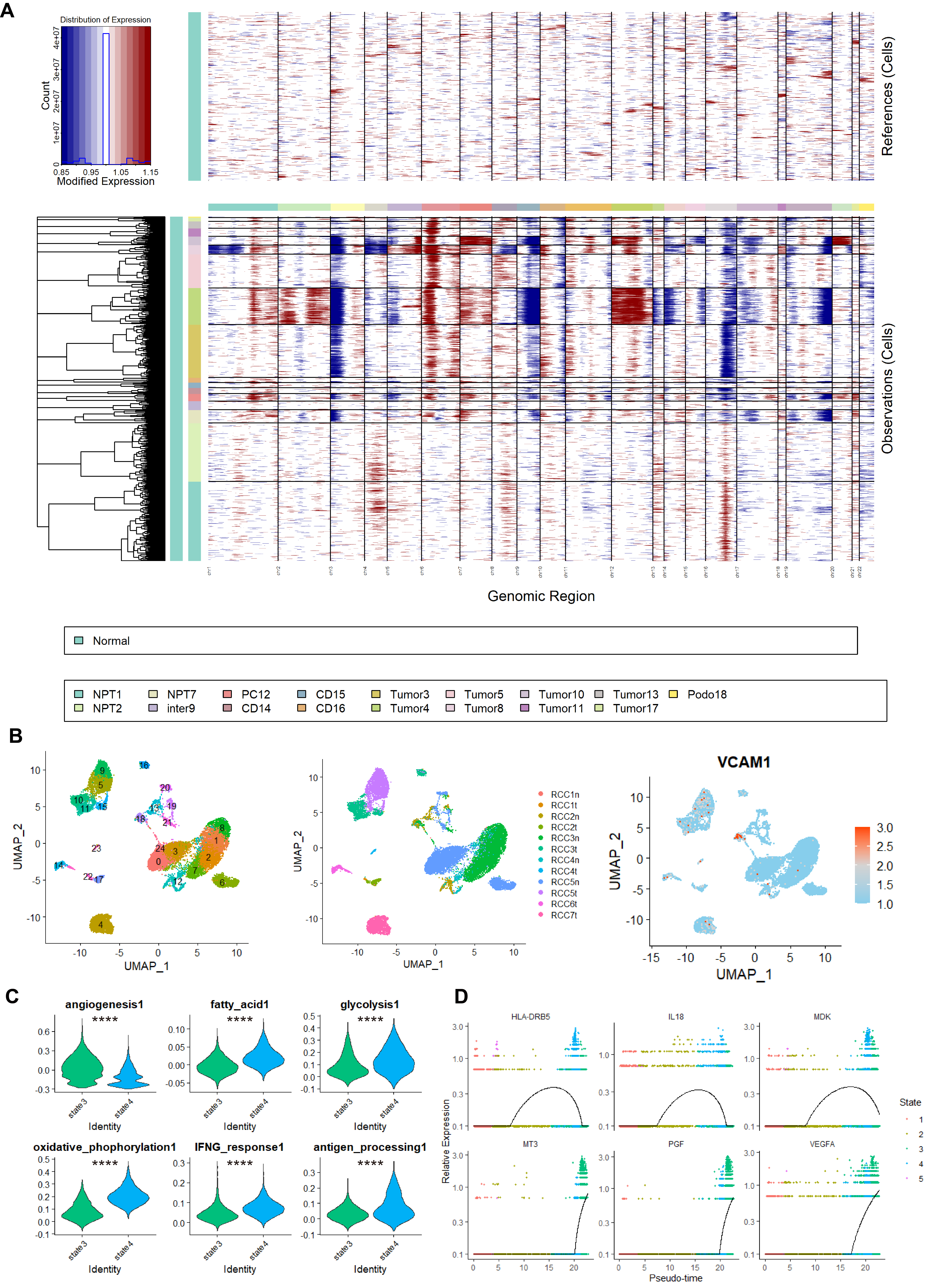

Supplement: Supplementary file 2 — Figure S2 (A) infer CNV results for all epithelial clusters with normal proximal tubule cells (NPT) cluster 0 and 6 as normal reference. PC: principal cells, CD: collecting duct cells, Podo: podocytes. inter9 stands for an intermediate cell cluster. (B) Left, Subclustering of all epithelial cells with a higher resolution, which were divided into 25 clusters. Cluster 18 stands for VCAM1+ cells. Middle, sample origins for epithelial cells. Right: VCAM1 expression profiles in epithelial cells. (C) Comparison of gene signature scores between state 3 and state 4 cells. ****stands for that p‐value of students’ t‐test was less than .0001. (D) Antigen presentation related genes HLA‐DRB5, IL18 and MDK were upregulated in state 4 cells, while angiogenesis related genes VEGFA and PGF, and hypoxia induced gene MT3 were upregulated in state 3 cells. [file CTM2-12-e713-s004.jpg]

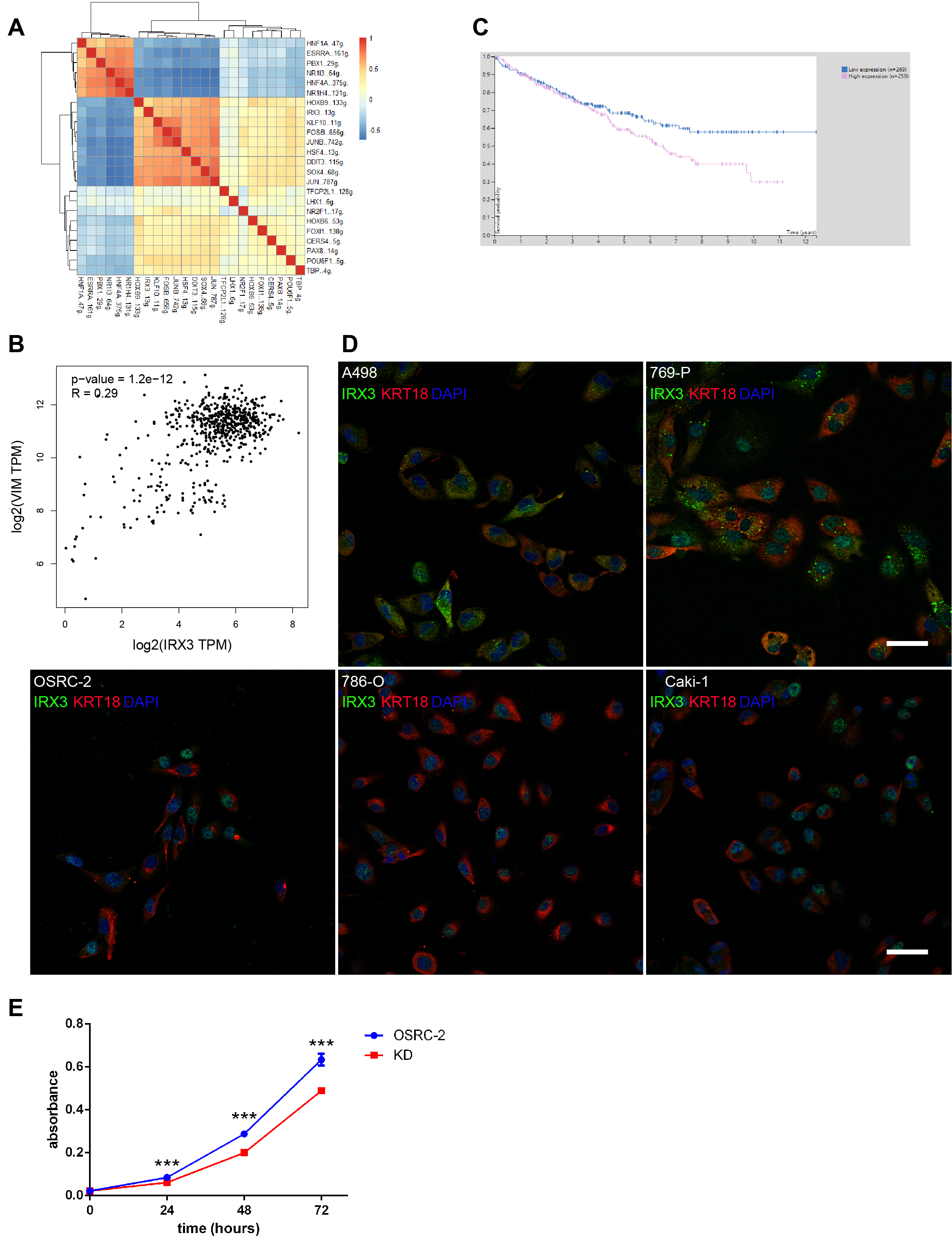

Supplement: Supplementary file 3 — Figure S3 (A) Correlation analysis of transcription factor regulons. Co‐upregulated regulons clustered together. (B) IRX3 and EMT gene VIM had correlation in TCGA cohort. (C)Overall survival curve of IRX3 in TCGA cohort containing 528 ccRCC patients. p‐score = .048. This figure was obtained from https://www.proteinatlas.org/ENSG00000177508‐IRX3/pathology/renal+cancer/KIRC. (D) Immunofluorescence staining results of 5 ccRCC cell lines. IRX3 located mainly in cytoplasm for A498 and 769‐P, whereas IRX3 mainly located in nuclei of OSRC‐2, 786‐O and Caki‐1. Scale bar: 20μm. (E) Viability validation with CCK8. IRX3 knockdown cell lines were obviously weaker than wildtype. [file CTM2-12-e713-s006.jpg]

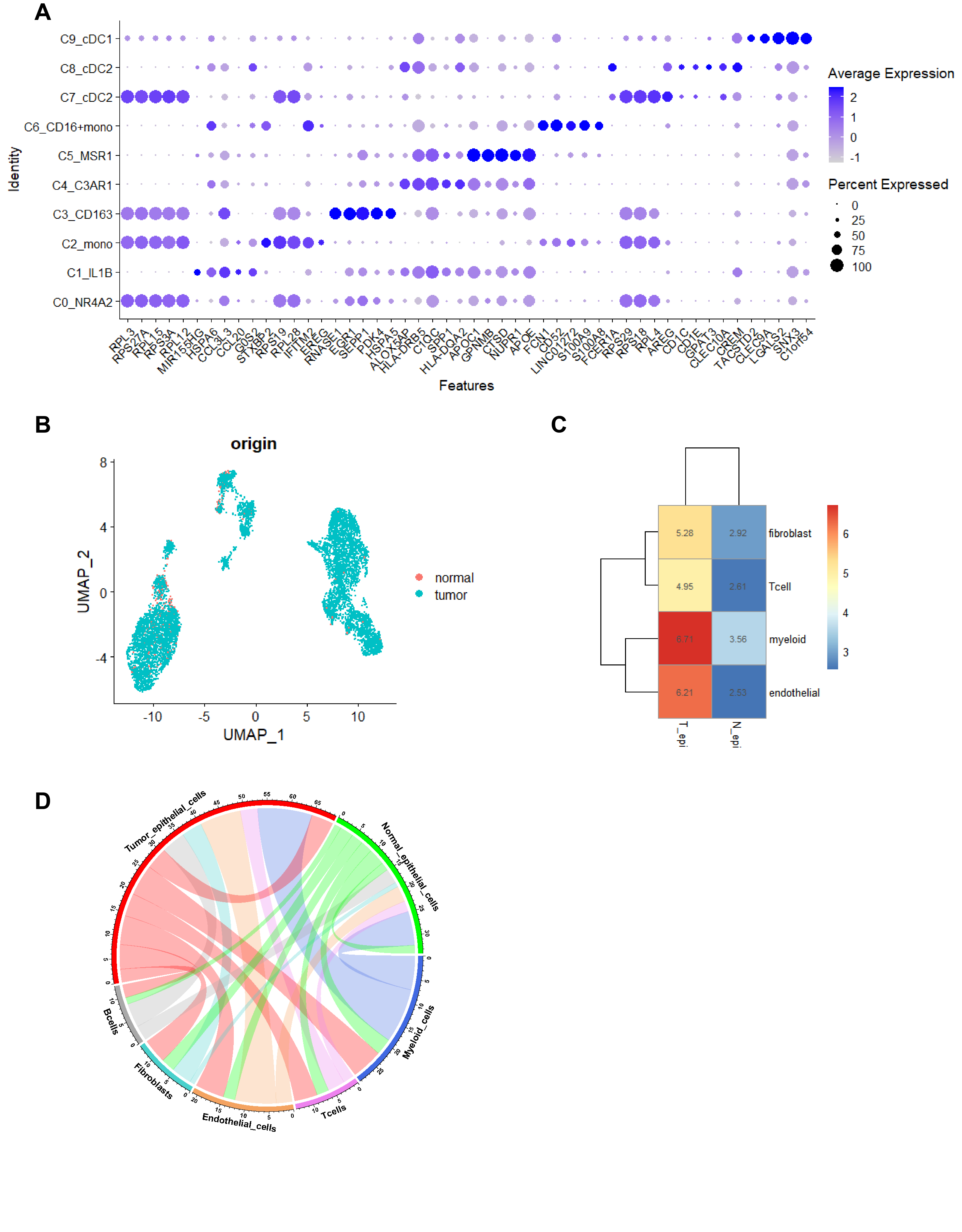

Supplement: Supplementary file 4 — Figure S4 (A) Top 5 genes and expression levels in myeloid clusters. (B) Sample origins for all the myeloid cells. (C) Interaction strength between different cells in tumour versus normal samples. T_epi: tumour epithelial cells, N_epi: normal epithelial cells. (D) Circos plot indicates the interaction strengths among tumour epithelial cells versus other cell types, and normal epithelial cells versus other cell types. [file CTM2-12-e713-s002.jpg]
