# Supplementary material for: Single cell analysis reveals intra‐tumour heterogeneity, microenvironment and potential diagnosis markers for clear cell renal cell carcinoma
Source: Clin Transl Med. 2022 May 23;12(5):e713. doi: 10.1002/ctm2.713 (PMC9126499; doi:10.1002/ctm2.713)
Supplement: Supplementary file 12 — Supporting information [file CTM2-12-e713-s012.docx]

**Materials and Methods**

**1 Patient samples**

All patients included in the study underwent nephrectomy at the Renji Hospital (Table S1). Tissue samples were obtained after signing informed consent and in compliance with Renji Hospital Ethics Committee approved protocols (RA-2020-350).

**2 Data and code availability**

Single cell RNA sequencing raw data can be obtained from the GEO with accession number GSE156632. Codes or softwares followed the official tutorials.

**3 Single-cell suspension**

Tumor or normal tissue was minced in 750µl DMEM with collagenase IV (100µg/ml, Gibco) in 1.5ml EP tube, another 750ul DMEM with collagenase was added and incubate at 37℃ for 30 min. Tubes were spun at 350 rcf for 5 min and the supernatant was removed. 1mL DMEM containing DNase I (100ug/ml, Beyotime) was added into the tube and the pellet was resuspended. The suspension was filtered with 70µm strainer and the filtrate was spun at 350rcf for 5 min. The pellet was resuspened with 400µl DMEM. 200µl Lympholyte-H separation (Cedarlane) was added to the bottom of the tube and spun at 800rcf for 20 min. Transfer the supernatant to a new tube, add DMEM as much as possible. The tube was spun at 800rcf for another 20 minutes and the supernatant was removed. Pellet was resuspended with 100ul-200ul PBS to get the single cell suspension.

**4 Data preprocessing, quality control**

Illumina bcl2fastq (version 2.19.1) was used to transform the raw data to FASTQ file. Then files were aligned to GRCH38. Genes-cells matrix was got after Cell Ranger (version 2.2.0) treatment. Seurat package (version 4.0.5) was used to analyze the gene expressing matrix. Cells with less than 500 genes and mitochondrial gene percentage more than 10% were filtered out. Mitochondrial genes had been excluded before downstream analysis. DoubletFinder_v3 was used to remove the doublets and evaluate whether the data contained any populations with doublets according to cell type-specific markers’ expressing profiles.

**5 Normalization, dimensionality reduction, clustering, batch effect removing, and visualization**

For normalization, SCTransform in Seurat (version 4.0.5) was used. PCA was used to reduce the dimensionality of the single cell RNA sequencing data. Graph-based clustering was performed and RunUMAP (dims=1:30, resolution=0.6, or 1.2) was used for visualization. For batch effect removing, harmony (version 0.1.0) function was run after PCA.

**6 Cell type definition**

Representative markers were used to assign each cluster into a cell type: epithelial cell (KRT8, KRT18, CD24, EPCAM), myeloid cell (CD68, CD14, CSF-1R), T cell (CD2, CD3D, CD3E, CD3G), endothelial cell (ENG, PECAM1, VWF, CDH5), fibroblast (TAGLN, RGS5, MYL9, ACTA2), B cell (CD79A, CD79B, MS4A1).

**7 Differentially expressed genes (DEGs) analysis**

FindMarkers or FindAllMarkers function in Seurat (version 4.0.5) was used. All the genes were [prese](javascript:;)ted expressing in more than 10% of cells in either of these two clusters evaluated and the expressing difference at natural log scale was more than 0.1.

**8 InferCNV**

InferCNV (version 1.2.1) was used. For epithelial cells, object was the raw_counts data, normal proximal tubule cells were set as reference, the gencode_v19_gene_pos.txt was gene_order_file, cut off = 0.1.

**9 RNA velocity analysis**

scVelo (version 0.2.3) in Python was used for prediction. Loom files were read into the scvelo python with scv.read function, then count matrix was obtained. The matrix was normalized to the median of overall RNAs through cells. Top 3000 highly variable genes were chosen. Then recover_dynamics was run. At last figures were shown in UMAP with streamlines indicating the transition tendency.

**10 NMF (0.23.0) analysis**

To investigate tumor specific transcriptional programs, 70 sample modules were obtained through the similarity matrixes’ optimal factors. Top 30 genes for every module were chosen to be the representatives of module features. Modules of all tumor cells were scored. Correlation analysis was performed and then heatmaps with modules containing correspondent scores were shown and finally 6 meta-programs were obtained. For each program, gene signature scores were averaged and the top 50 genes were selected to be the program’s signatures.

**11 SCENIC analysis**

pySCENIC (version 0.1.5) was used to infer the active transcription factors and the targets. GRNBoost was run to get the co-expressed modules. Motifs database of Homo Sapiens was obtained from https://pyscenic.readthedocs.io/en/latest/. Input gene matrixes were the raw counts. AUCell was run to get the score of regulon activities and then cell type specific regulons can be discovered.

**12 Calculation of cell type abundance with CIBERSORTx**

After subclustering of myeloid cells or T cells, the differentially expressed genes (DEGs) of every cluster were found through performing Seurat’s FindAllMarkers function, and then the mean expressing values of DEGs in every cluster were calculated. The matrix that we obtained was a gene signature file. The bulk expression file and clinical file were obtained from https://portal.gdc.cancer.gov/. Log in the CIBERSORTx website and upload the gene signature file and the expression matrix, the calculating result was the inferred proportion of different cell types.

**13 Cell-cell interaction analysis with CellPhoneDB v2**

Cells from the 12 samples were clustered and cell types were determined. Iterations were set to 1000 and threads were set to 30. Non log-converted counts were used as the expressing levels for ligand and receptor genes. Ligand-receptor pairs with P values less than 0.05 were returned.

**14 Immunofluorescence staining**

Tumor and para-tumor tissues were treated using 4% paraformaldehyde at 4℃ overnight and embedded. Paraffin sections were treated with xylene twice for 10 min and rehydrated with graded ethanol. Slides were washed twice. Retrieval was performed with 0.25M pH9.0 Tris-EDTA retrieval buffer. Sections were then rinsed with PBS and incubate with blocking buffer for 30 min, which was 5% donkey serum in PBS containg 0.3% Triton-X100. Slides were treated using primary antibodies [anti-IRX3 (Abcam ab247145, 1:50 dilution), anti-KRT18 (ProteinTech 66187-1-Ig, 1:50 dilution), anti-C3 (ProteinTech 66157-1-Ig, 1:100), anti-C3AR (Abcam ab59554, 1:20), anti-LGALS9 (Cloud-Clone PAA309Hu01, 1:100), anti-CD47(ProteinTech 66304-1-Ig, 1:100)] 8 hrs. at 4°C. Slides were then rinsed using PBS for 5 min ×3 times and treated with secondary antibodies (Alexa Fluor 594-conjagated with HRP or Alexa Fluor 488-conjagated with HRP, Jackson Lab) for 1h at RT. Sections were rinsed for 5 min ×3 times. The slides were stained with DAPI and observed under Leica DM2500 or Leica TCS SP5 inverted confocal.

**15 Wound healing**

3×10^5^ OSRC-2 wild-type or knockdown cells were seeded into 6-well plate separately. After reaching the confluence, straight lines were scratched in well plate bottom with the help of a ruler. The wells were washed gently and new medium were added. Photos were taken and time point was recorded as 0h. After incubating at 37℃ for 20 hours, second batch photos were taken. The healing areas were calculated by Image J.

**16 Migration and invasion**

2×10^4^ OSRC-2 wild-type or IRX3-knockdown cells were seeded into the superior well of transwell plates in 100µl medium without FBS. The lower wells were filled with 500µl 1640 medium containing 10% FBS separately. After incubating at 37℃ for 40 hrs., upper wells were washed gently and fixed for 10 min before staining in crystal violet for 10 min. Then the upper wells were washed in PBS, and the cells in the upper layer of upper wells were wiped with absorbent cotton gently. Upper wells were washed with PBS again and photographed under Olympus CKX53 microscope. For invasion experiments, before adding cells suspension, 100µl 1:2 diluted matrigel was added into the bottom of each upper well and after drying at 37℃ for 2 hours, 100µl cell suspension in 1640 medium without FBS was added into each well. After incubating at 37℃ for 24 hours, upper wells were washed and fixed before staining with crystal violet.

**17 Viability validation with CCK8**

For each well of 96 well plates, 1×10^3^ OSRC-2 wild-type or IRX3- knockdown cells were seeded into 90µl complete medium. For each cell line, 6 repeats were set and medium was set as blank control. Plates were incubated at 37℃ for 0, 24, 48, 72hours. Before reading, 10µl CCK8 was added into the each well. The plates were shaken gently and incubate at 37℃ for 1 hour, then the absorbance at 460nm were recorded with microplate reader.
